# Multidimensional exploration of the relationship between gut microbiota and colorectal cancer: focus on clinical tumorigenesis and treatment

**DOI:** 10.3389/fimmu.2026.1807247

**Published:** 2026-05-07

**Authors:** Haiyu Zhang, Ke Zhang, Jichuan Liu, Hao Luo

**Affiliations:** 1 Department of Transfusion, Sichuan Clinical Research Center for Cancer, Sichuan Cancer Hospital & Institute, Sichuan Cancer Center, University of Electronic Science and Technology of China, Chengdu, Sichuan, China; 2 Department of Clinical Laboratory, Sichuan Clinical Research Center for Cancer, Sichuan Cancer Hospital & Institute, Sichuan Cancer Center, University of Electronic Science and Technology of China, Chengdu, Sichuan, China

**Keywords:** colorectal cancer, gut microbiota, research progress, treatment strategies, tumorigenesis

## Abstract

The gut microbiota has received considerable attention in the field of colorectal cancer (CRC) research in recent years. In this review, we have explored the multifaceted relationship between the gut microbiota and CRC progression and treatment. The composition, distribution, and normal physiological functions of the gut microbiota have been summarized, along with the association between gut dysbiosis and CRC based on the body of evidence from animal experiments and clinical studies. In addition, we have discussed the mechanisms through which specific microbial configurations or microbiota-derived metabolites may contribute to colorectal carcinogenesis, including genotoxic effects, inflammation, and immune dysregulation. The impact of the gut microbiota on the efficacy of chemotherapy, radiotherapy, and immunotherapy, and new treatment strategies based on the gut microbiota, such as probiotic intervention, prebiotic application, and fecal microbiota transplantation have also been explored. Despite some promising outcomes, the specific carcinogenic microorganisms have not been identified, and it is challenging to distinguish association from causation, determine the influence of individual differences, and translate the research to clinical applications. In the future, more rigorous longitudinal studies, gnotobiotic models with defined microbial communities, and mechanistic interventional studies are needed to strengthen causal inference, and provide practical guidance for CRC prevention and treatment. Beyond summarizing reported associations, this review proposes a microbiota-immune-metabolism-therapy axis by integrating tumorigenic mechanisms, immune contexture, and treatment responsiveness into a single translational framework.

## Graphical Abstract

**Figure:**
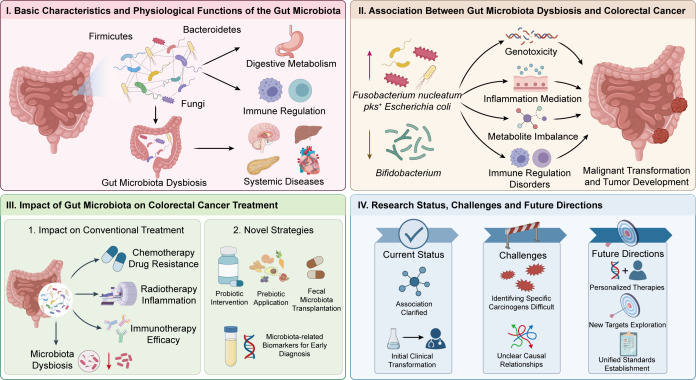
This graphical abstract shows gut microbiota’s basic traits, its association with colorectal cancer, impact on cancer treatment, and current research status, challenges and future directions.

## 1 Introduction

The gastrointestinal tract is not only the site of digestion and nutrient absorption, but also a complex and dynamic micro-ecosystem. More than a thousand species of bacteria, fungi, protozoa, viruses, and other microorganisms inhabit the gastrointestinal tract (1–3), and the total number of microbial cells exceeds 100 trillion, which is 10 times the total number of human cells. Moreover, these microbes form a close and dynamically balanced micro-ecological network that has a profound impact on human health (4–6). The gut microbiota secrete specific enzymes that break down macronutrients like indigestible complex carbohydrates, proteins, and fats into small molecules that are easily absorbed by the body, thus greatly improving the utilization rate of nutrients (7–9). *Bacteroides* and *Ruminococcus* are the key gut bacteria involved in metabolizing dietary carbohydrates like starch, pectin, cellulose, etc., as well as host-derived mucin sugars. Furthermore, the gut microbiota and the intestinal mucosa form the first line of defense against the invasion of foreign pathogens (10–12). The commensal bacteria occupy ecological niches in the intestine and compete with pathogens, thereby inhibiting colonization by pathogenic microorganisms. In addition, the gut microbes also modulate the development and maturation of T cells, B cells, and innate immune cells in the intestine through certain metabolites, and maintain the balance between immune tolerance and inflammation.

In recent decades, the relationship between the gut microbiota and the occurrence and development of various diseases, particularly intestinal tumors, has been elucidated in greater detail. Colorectal cancer (CRC) is among the most prevalent malignancies worldwide, with consistently high incidence and mortality rates (13–15). According to the latest global cancer statistics released by the International Agency for Research on Cancer (IARC) of the World Health Organization (WHO), the incidence of CRC has risen significantly in China in recent years, with 550, 000 new cases diagnosed each year. Furthermore, studies increasingly show that gut dysbiosis is closely associated with the occurrence, progression, and treatment response of intestinal tumors (16–18). However, as the currently available human data is largely observational, a causal relationship has not been established. A greater understanding of the molecular mechanisms, signaling pathways, and microbe-host interactions involved in colorectal carcinogenesis is crucial for developing more effective diagnostic and treatment strategies.

In this review, we have proposed a microbiota-immune-metabolism-therapy axis for CRC wherein gut dysbiosis triggers immune remodeling and metabolic reprogramming in the intestine, which in turn influence the therapeutic responsiveness. Through this perspective, tumor initiation, immune microenvironment remodeling, microbial metabolites, and treatment response can be integrated into a more coherent translational model that links upstream ecological dysbiosis with epithelial injury, immune remodeling, metabolic signaling, and clinically relevant heterogeneity in treatment response. In addition, the review explicitly distinguishes associative human evidence from mechanistic support in experimental systems, thereby clarifying where the literature is sufficiently coherent for translational inference and where it remains primarily hypothesis-generating.

## 2 Overview of the gut microbiota

### 2.1 Composition and distribution of the gut microbiota

**Figure 1:**
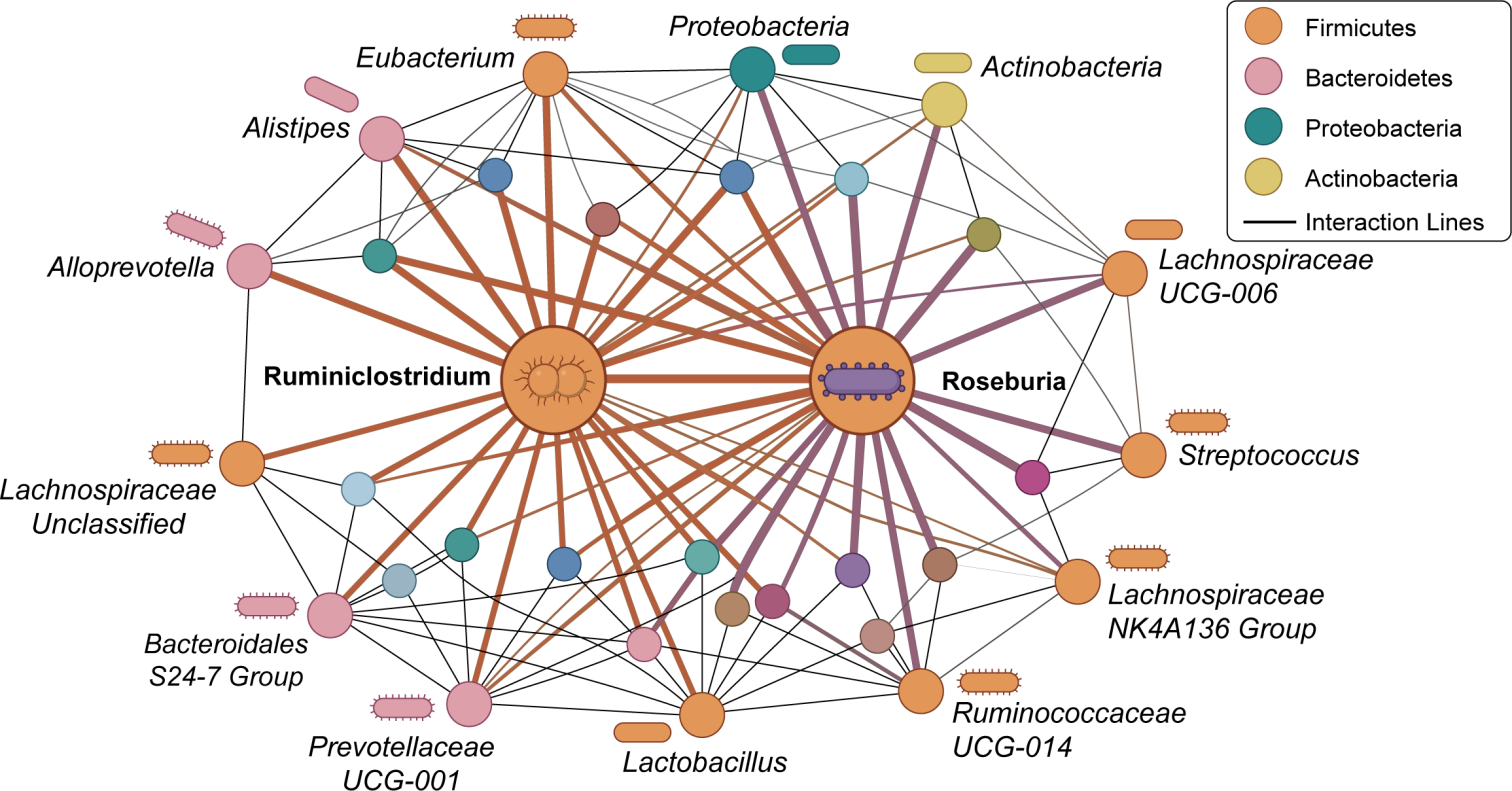
Ecological network of the gut microbiota. *Ruminiclostridium* and *Roseburia*, members of the phylum Firmicutes, interact most with the other bacteria.

The gut microbiota is a complex community of bacteria, fungi, archaea, and viruses, and the number of bacterial species exceeds 1000. From a taxonomic perspective, the intestinal bacteria mainly belong to four phyla: Firmicutes (*Bacillus*, *Clostridium*, and *Lactobacillus*), Bacteroidetes (*Bacteroides*), Actinobacteria (Bifidobacterium), and Proteobacteria (*Escherichia coli*) *(*19–21). The schematic diagram of the gut microbiota ecological network is shown in **Figure 1**.

The abundance and composition of the gut microbes show distinct spatial distribution patterns that are closely related to the physiological differences across the gastrointestinal tract (21–23). The acidic environment (pH 1-3) and relatively high oxygen content of the stomach are unfavorable for most bacteria and impede bacterial reproduction. Therefore, the bacterial load in the stomach is low, ranging from 10³-10^4^ bacteria per milliliter, and mainly includes acid-resistant, aerobic Gram positive bacteria such as *Helicobacter pylori* and *Lactobacillus (*24–26). The composition of the duodenal microbiota is similar to that of the stomach, and the bacterial load is 10³-10^4^ per milliliter of intestinal contents. In addition to some gastric bacterial species, the duodenum also harbors *E. coli* and other anaerobic bacteria. While the bacterial load in the jejunum remains at 10³-10^4^ per milliliter, the dominant species are Gram positive aerobic bacteria, including *Lactobacillus*, *Streptococcus*, and *Staphylococcus (*27–29). On the other hand, the ileum has a high bacterial load of 10^8^ bacteria per milliliter, and a higher proportion of anaerobic bacteria relative to the aerobic bacteria, with *E. coli* being among the most ubiquitous species (30, 31). The colon has the highest bacterial load with up to 10¹² bacteria per milliliter, and more than 98% of them are obligate anaerobic bacteria, mainly *Bacteroides*, *Bifidobacterium*, *Eubacterium*, anaerobic Gram positive cocci such as *Enterococcus* (32–34), *Peptococcus* and *Peptostreptococcus*, and different species of Enterobacteriaceae.

### 2.2 Normal functions of the gut microbiota

**Figure 2:**
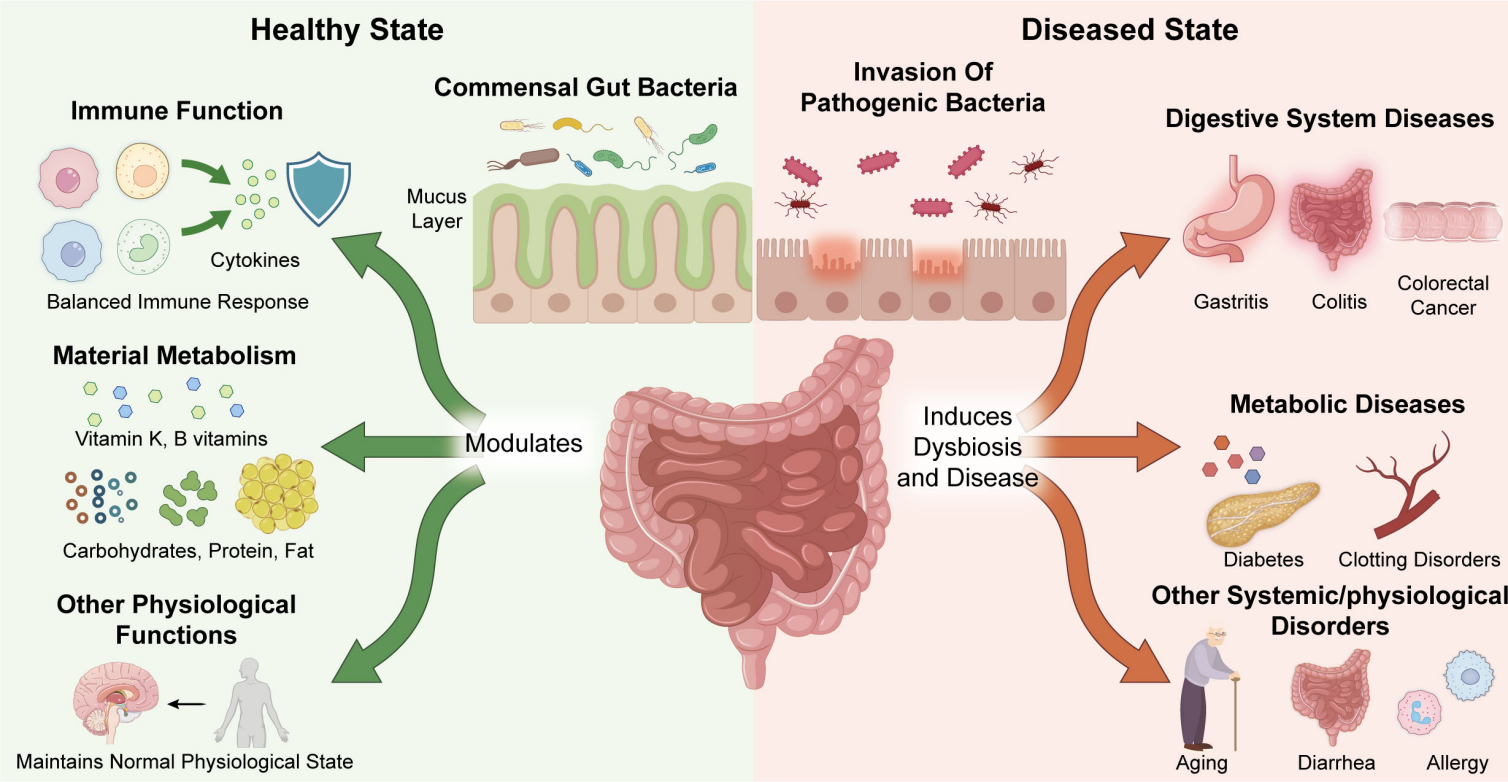
The intestinal flora affects immune, metabolism and other functions of the host. Gut dysbiosis is associated with gastrointestinal, metabolic, and other systemic diseases.

The gut microbiota plays an indispensable role in the digestion process. They can decompose macronutrient substances that cannot be directly utilized in the human gastrointestinal tract – such as indigestible complex carbohydrates, proteins, and fats – into small-molecule nutrients (34–36) for easy absorption, thereby improving nutrient utilization. Furthermore, some strains can use these degradation products to synthesize substances necessary for their own growth, as well as metabolites that are beneficial to the host, such as vitamins and bioactive peptides (37–39). Gut microbes promote fat absorption by secreting lipases and other enzymes that emulsify and decompose fat, and by regulating the composition, metabolism, and function of bile acids (40–42). Given the role of intestinal microorganisms in nutrient metabolism, it is not surprising that gut dysbiosis is linked to diseases like diabetes and colitis (43–45). The gut microbes also synthesize essential vitamins, such as vitamin K, vitamin B1, vitamin B2, vitamin B6, vitamin B12, and folic acid, which cannot be produced by the human body. Vitamin K is crucial for blood coagulation, and its deficiency can lead to excessive bleeding and severe internal hemorrhage (46–48). Various gut bacteria contribute to vitamin K synthesis, mainly *Bifidobacterium* and *E. coli*. Likewise, diverse bacterial species are involved in the synthesis of B-group vitamins, which play key roles in energy metabolism, nucleic acid synthesis, and nervous system function. For instance, *Lactobacillus* and *Bifidobacterium* strains synthesize folate and vitamin B2, and vitamin B12 is primarily produced by *Lactobacillus* and *Propionibacterium* (49–51). The involvement of the gut microbiota in the immune, metabolic, and other physiological functions of the host is shown in **Figure 2**.

## 3 Gut microbiota and colorectal tumorigenesis

### 3.1 Incidence status and trends of CRC

CRC is the most common malignancy of the digestive system and poses a serious threat to human health worldwide. According to IARC data, CRC ranked third among all malignancies in terms of incidence, and the second in terms of mortality worldwide in 2020, with as many as 1.93 million cases and 940, 000 deaths recorded that year (52–54). On average, more than 5000 people are diagnosed with CRC every day globally, and more than 2500 deaths occur daily due to CRC.

At the global level, the incidence of CRC shows a long-term upward trend. In the past few decades, with the improvement of industrialization levels, changes in lifestyle, and the deepening of aging, the incidence of CRC has been increasing (55–57). Especially in countries and regions with rapid economic development, such as East Asia and Southeast Asia, the incidence of CRC has increased significantly. Globally, from 1990 to 2019, the number of new CRC cases increased from 842, 100 to 2.17 million, a 1.6-fold increase; the number of death cases increased from 518, 100 to 1.09 million, a 1.1-fold increase. The burden in East Asia is particularly severe (58–60). In 2019, the number of new cases reached 637, 000, and the number of death cases reached 276, 000. In recent years, due to factors such as the improvement of residents’ living standards, the westernization of the diet structure, and changes in lifestyle, the incidence and mortality of CRC have both shown an upward trend in China (61–63). Data from the National Cancer Center of China shows that in 2020, the number of new CRC cases in China was approximately 555, 000, and the number of death cases was 286, 000. The incidence and mortality ranked second and fifth respectively among all malignant tumors. Geographically, the incidence of CRC is slightly higher in urban areas, and the increase in mortality in rural areas is more prominent. In addition, the age of onset of CRC is getting younger. In the past, it mostly occurred in the elderly over 60 years old (64–66). In recent years, the number of patients aged 40-50 years has been increasing, and there are even young patients under 30 years old, which poses a great challenge to the prevention and treatment of the disease.

### 3.2 Evidence of the association between gut dysbiosis and CRC

Fecal or intestinal microbiota from heathy or tumor-bearing mice have been transferred into germ-free recipients – a procedure known as fecal microbiota transplantation (FMT) – to explore whether microbial communities can modify susceptibility to colorectal tumorigenesis. Compared to germ-free controls, recipient mice colonized with these microbiota develop more or larger tumors under carcinogen exposure (such as azoxymethane, AOM) or in genetically susceptible settings (such as *APC* gene mutation) (67, 68). These findings support a contributory role of the microbiota in experimental colorectal tumorigenesis, although they should be interpreted as evidence from controlled model systems rather than direct proof of causality in humans. Studies using gnotobiotic mice colonized with defined strains or microbial consortia have shown that *Fusobacterium nucleatum* can increase tumor burden under specific experimental conditions. *F. nucleatum* binds to E-cadherin on the surface of intestinal epithelial cells through its surface FadA adhesin and activate the Wnt/β-catenin signaling pathway, thereby promoting cellular proliferation and survival (69–71). In addition, *MyD88*-knockout mice have a significantly higher risk of developing AOM-induced intestinal tumors compared to wild-type mice. Myeloid differentiation primary response 88 (MyD88) is the key adaptor protein of the Toll-like receptor (TLR) signaling pathway, and the loss of *MyD88* has been associated with impaired immune response to the gut microbiota (72, 73). Consistent with this, the gut microbiota of the *MyD88*-knockout mice showed significant changes, mainly characterized by a marked increase in the abundance of *Proteobacteria*, and a concomitant decrease in *Firmicutes* and *Bacteroidetes*. These observations suggest that host-microbiota immune interactions may influence the susceptibility to colorectal tumorigenesis (74–76), with the MyD88 pathway playing a key modulatory role.

Numerous clinical studies have also reported reproducible associations between gut dysbiosis and CRC through comparative analyses of the gut microbiota in CRC patients and healthy individuals. While these datasets are valuable for defining disease-associated microbial patterns, they are primarily derived from cross-sectional or case-controlled studies, and therefore cannot determine whether dysbiosis is the initiating event or the outcome of colorectal carcinogenesis, or if it merely reflects confounding factors like diet, antibiotic exposure, inflammation, or treatment. Accordingly, longitudinal studies with serial sampling before adenoma formation, during early neoplastic transformation, and after treatment are needed to clarify temporal sequence and strengthen causal inference. In addition, gnotobiotic models colonized with defined strains or synthetic microbial communities provide a powerful experimental platform to test whether candidate organisms are sufficient for tumor initiation or progression, and to dissect microbe-host mechanisms under controlled conditions.

### 3.3 Mechanisms linking gut dysbiosis to the occurrence of CRC

**Figure 3:**
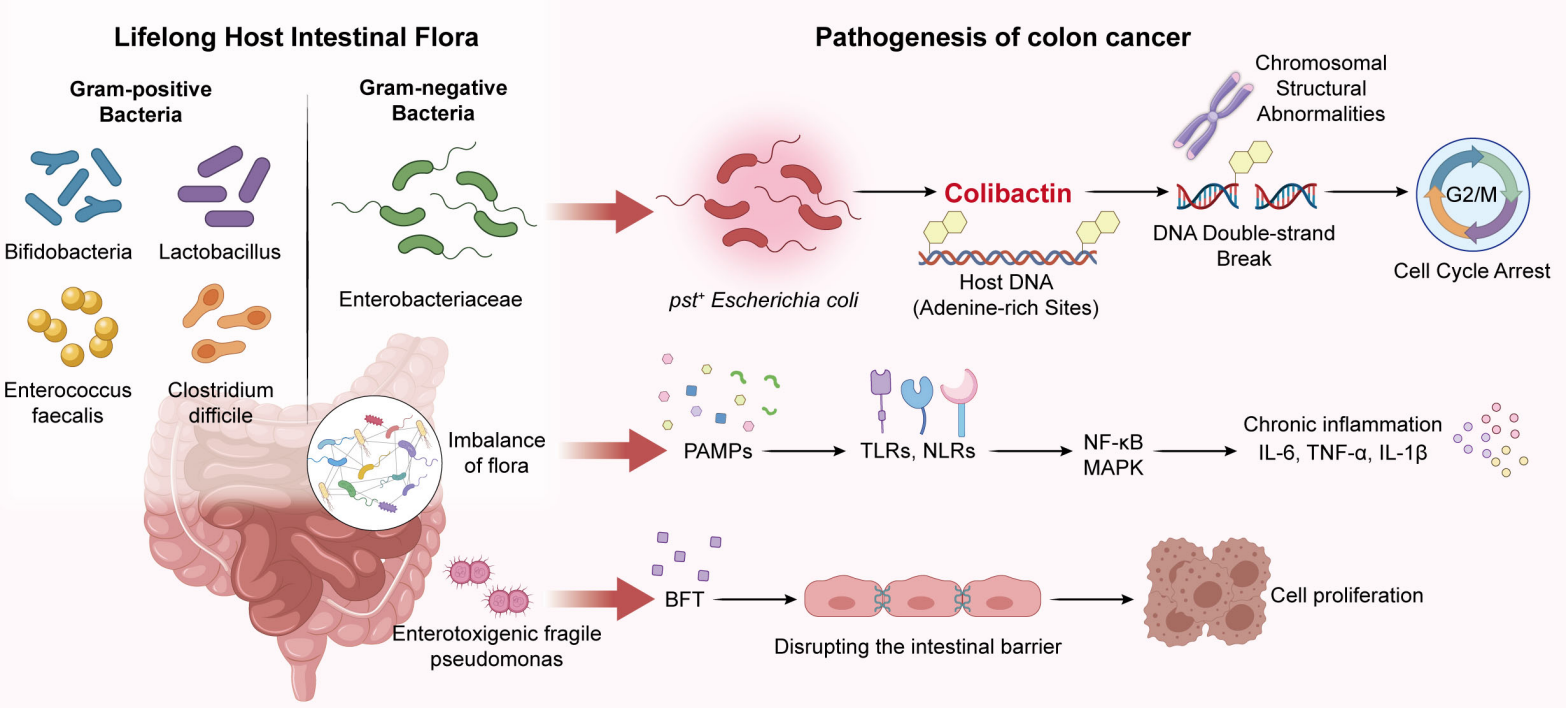
This schematic shows how lifelong gut microbiota imbalance drives colon carcinogenesis via three interconnected mechanisms: *pks^+^ E. coli*-derived colibactin induces DNA damage and cell cycle arrest, dysbiotic bacteria trigger chronic inflammation through TLR/NLR-NF-κB/MAPK signaling, and enterotoxigenic *B. fragilis* disrupts the intestinal barrier to promote excessive cell proliferation.

Pks+ *E. coli* are gut-colonizing bacteria characterized by the presence of the *pks* gene island, and have been identified as a potential risk factor in CRC. The *pks* island encodes enzymes involved in the synthesis of colibactin, a DNA alkylating toxin that induces inter-strand cross-links, double-strand breaks, and chromosomal instability in the host epithelial cells, which are conducive to malignant transformation (**Figure 3**) (77–79). In fact, colibactin has been shown to promote colorectal carcinogenesis in experimental models by causing mutations in the cancer driver genes. Furthermore, pks+ *E. coli* can penetrate the intestinal epithelial barrier by producing virulence factors, and directly adhere to the host cells to cause genotoxic damage. Consistent with the above findings, the prevalence of pks+ *E. coli* is significantly higher in CRC patients compared to healthy individuals, and its abundance in the colorectal tumors correlates with tumor aggressiveness and prognosis. However, these observations are indicative of clinical association and a plausible mechanism, and do not establish a causal role of pks+ *E. coli* in colorectal carcinogenesis (80–82). Enterotoxigenic *Bacteroides fragilis* has also been implicated in colorectal tumorigenesis. The *B. fragilis* toxin (BFT) cleaves the tight-junction proteins between intestinal epithelial cells, which impairs epithelial barrier integrity, and facilitates the passage of bacteria and microbial products across the mucosa. In addition, BFT have been shown to enhance the proliferation of intestinal epithelial cells and suppress apoptosis, trigger inflammatory responses (83–85), and activate oncogenes downstream of the Wnt/β-catenin signaling pathway. While these findings support mechanistic plausibility in experimental systems, the current evidence from human studies is more consistent with association rather than causation across the broader CRC population. Gut dysbiosis disrupts the balance between symbiotic bacteria and the opportunistic pathogens in the intestine, which may favor a chronic inflammatory milieu. Bacterial pathogen-associated molecular patterns (PAMPs) like lipopolysaccharide and flagellin can be recognized by pattern-recognition receptors such as TLRs and NOD-like receptors (NLRs) on the surface of intestinal epithelial cells and immune cells (86–88). The interaction of these receptors to PAMPs activates the NF-κB and MAPK pathways, resulting in the sustained release of inflammatory factors such as interleukin (IL)-6, tumor necrosis factor α (TNFα), and IL-1β, which can support tumor initiation and progression. While this sequence has been demonstrated in experimental models, it cannot be viewed as a universal causal pathway of CRC.

The gut microbiota also contributes to colorectal tumorigenesis by modulating the tumor immune microenvironment (TIME). The colorectal TIME is characterized by a complex network of epithelial cells, stromal cells, dendritic cells (DCs), macrophages, neutrophils, myeloid-derived suppressor cells (MDSCs), and lymphocyte subsets, and microbial cues can influence both the composition and the functional state of these immune populations. While intra-tumoral CD8+ T cells are associated with anti-tumor surveillance, the enrichment of FOXP3+ regulatory T cells (Tregs), suppressive myeloid populations, and dysfunctional antigen-presenting cells (APCs) tends to weaken effector immunity through inhibitory cytokines, metabolic restriction, and checkpoint-linked pathways. Under dysbiotic conditions, microbial products and metabolites may skew myeloid cell polarization, alter DC maturation and antigen presentation, and favor the recruitment or persistence of immunosuppressive populations, thereby sustaining an inflammatory but tumor-permissive immune niche. Recent single-cell RNA sequencing and spatial transcriptomic analyses have demonstrated that the immune landscape of colorectal tumors consists of distinct functional states, including cytotoxic versus exhausted CD8+ T cell programs, suppressive Treg states, heterogeneous macrophage and DC populations, and spatially restricted ligand-receptor interactions among epithelial, stromal, and immune compartments. These high-resolution approaches can identify the cellular programs through which microbial dysbiosis may be linked to T cell dysfunction, immune exclusion, or myeloid-mediated suppression. Although direct causal assignment of individual microbial taxa to specific immune cell states remains incomplete, single-cell and spatial technologies provide a framework for connecting microbiota signatures with the cellular and functional architecture of the colorectal TIME.

## 4 Gut microbiota and CRC treatment

### 4.1 Overview of the current CRC treatment methods

Surgery is an effective approach to achieve cure in early-stage CRC patients, surgery is an effective way to achieve cure. The surgical method is selected on the basis of tumor location, shape, size, and invasion range. On the other hand, chemotherapy is routinely used for the treatment of middle- and late-stage CRC in combination with surgery or radiotherapy (77, 78, 89). The common chemotherapy drugs used in CRC regimens include fluorouracil (5-FU), oxaliplatin, irinotecan, etc. These drugs have different mechanisms of action that target DNA synthesis, cell division, or metabolic processes. Chemotherapy can relieve symptoms and extend the survival of late-stage CRC patients (79–81). However, the non-specific effects of chemotherapy drugs on normal cells often trigger adverse reactions, including nausea, vomiting, hair loss, bone marrow suppression, and damage to liver and kidney functions. Radiotherapy uses high-energy X-rays, γ rays, etc. to irradiate the tumor site. The ionizing radiations induce DNA damage in the cancer cells and impair their proliferative ability, resulting in tumor cessation. In patients with locally advanced rectal cancer (82–84), preoperative radiotherapy can reduce the tumor mass and lower the tumor stage, improve the surgical resection rate and sphincter preservation rate, and lower the risk of local recurrence.

Immunotherapy has emerged as an important treatment modality in CRC as it aims to restore or augment anti-tumor immunity rather than directly targeting the tumor cells. The principal immunotherapeutic strategies for CRC include immune checkpoint blockade and adoptive cell-based approaches. Immune checkpoint inhibitors such as pembrolizumab and nivolumab target programmed cell death protein 1 (PD-1), programmed death-ligand 1 (PD-L1), and cytotoxic T-lymphocyte-associated antigen 4 (CTLA-4), and mitigate the signals that constrain T cell activation, proliferation, and effector function (90–92). However, checkpoint blockade does not induce anti-tumor immunity *de novo*; its efficacy depends on efficient presentation and priming of effector cells, which can be impaired by immunosuppressive populations. Adoptive transfer of ex vivo expanded cytokine-induced killer cells and chimeric antigen receptor T cells can overcome this limitation by providing effector cells capable of recognizing and eliminating tumor cells. Together, these approaches broaden the therapeutic landscape of CRC, while also underscoring the importance of the immune microenvironment in determining patient response.

### 4.2 Impact of gut microbiota on the efficacy of CRC treatment

#### 4.2.1 Impact on chemotherapy

Some intestinal bacteria synthesize enzymes that metabolize chemotherapy drugs into their therapeutically active forms. For instance, *E. coli* produce β-glucuronidase, which can convert SN-38 glucuronide, the inactive metabolite of irinotecan, into the active metabolite 7-ethyl-10-hydroxycamptothecin (SN-38), thereby enhancing the anti-tumor effect of irinotecan (85–87). The types and functional states of the gut microbiota can alter the metabolic rates and pathways of chemotherapy drugs. Accordingly, a dysregulated gut microbiota can adversely affect the metabolism of chemotherapy drugs, and alter their effective dosage and half-life, leading to suboptimal effects. Studies have shown that antibiotics-induced gut dysbiosis in mice alters the metabolism and excretion rate of chemotherapy drugs, resulting in increased accumulation that elevates toxic side effects (88, 93, 94).

**Figure 4:**
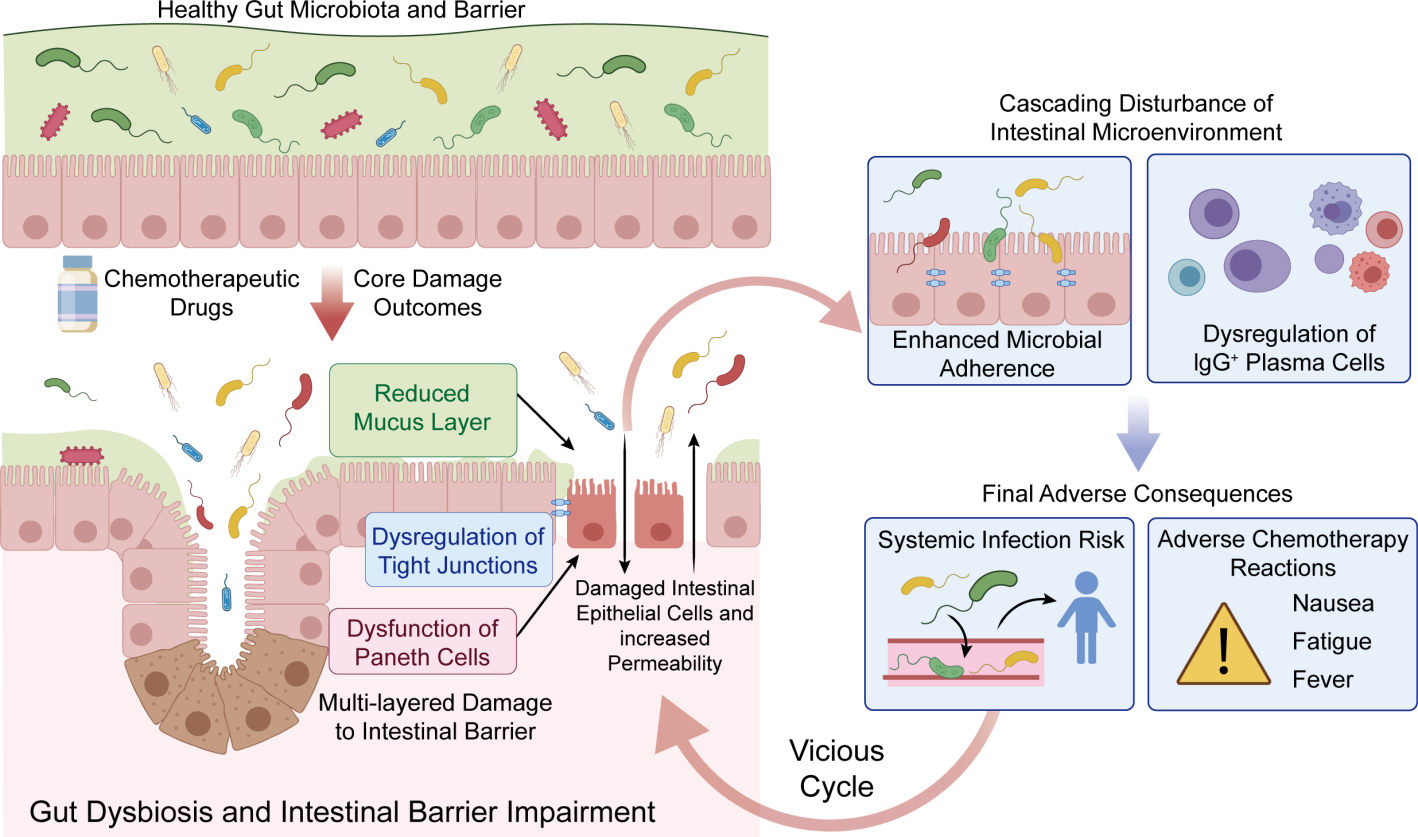
Interplay between chemotherapy drugs and the gut microbiota. Chemotherapeutic drugs can induce gut dysbiosis by impairing the intestinal epithelial barrier, which increases the risk of infection.

The gut microbiota can also alter the sensitivity of tumor cells to chemotherapy drugs by regulating the expression of proteins related to drug resistance. For instance, the presence of intestinal *F. nucleatum* has been linked to chemoresistance in CRC patients; mechanistically, *F. nucleatum* activates the TLR4-MYD88-NF-κB pathway and upregulates autophagy-related proteins, including pULK1, ULK1, and ATG, while downregulating the anti-apoptotic protein BIRC3 (95–97), resulting in tumor cells developing resistance to oxaliplatin and 5-FU. Likewise, γ-Proteobacteria may express the long isoform of bacterial cytidine deaminase, thereby metabolizing specific chemotherapeutic agents to less active forms. The gut microbiota can also influence the efficacy of chemotherapy drugs by regulating tumor metabolism and the tumor microenvironment. The short-chain fatty acids (SCFAs) produced by gut microbes during fermentation of dietary fibers can sensitize tumor cells to chemotherapeutic drugs through metabolic reprogramming, apoptosis induction, and modulation of oxidative stress. *Clostridium butyricum* supplementation in mice bearing colorectal tumors has been shown to increase the levels of butyric acid in tumor tissues and inhibit proliferation of malignant cells (98–100), and enhance the therapeutic effects of 5-FU. On the other hand, chemotherapeutic drugs can damage intestinal epithelial cells, alter intestinal permeability, and reduce the barrier function of epithelium, resulting in intestinal dysbiosis and an increased risk of infection (**Figure 4**).

#### 4.2.2 Impact on radiotherapy

During radiotherapy, the ionizing radiations cause direct damage to the intestinal mucosal barrier and increase permeability. The gut microbiota plays a crucial role in repairing mucosal damage, particularly probiotic strains such as *Bifidobacterium* and *Lactobacillus*. *Bifidobacterium* secretes mucin, which thickens the intestinal mucosa and enhances barrier function (90, 91, 101), whereas *Lactobacillus* accelerates mucosal repair by promoting the proliferation and differentiation of intestinal epithelial cells. Studies show that pre-treating mice with probiotics before radiotherapy can effectively reduce the radiation-induced intestinal damage, restore barrier function, and decrease bacterial translocation. Radiotherapy can exacerbate intestinal inflammation by inducing apoptosis of intestinal crypt cells and disrupting the microbiota structure. Pelvic radiotherapy leads to gut dysbiosis and induces secretion of the pro-inflammatory IL-1β, resulting in colon damage (102–104). The gut microbiota can mitigate radiation-induced inflammation and activate tissue repair by promoting secretion of anti-inflammatory cytokines such as IL-10 and transforming growth factor β (TGF-β) by the resident immune cells (92, 105, 106). Some studies indicate that SCFAs produced by the gut microbiota, mainly butyric acid and propionic acid, can reduce intestinal inflammation by inhibiting pro-inflammatory signaling pathways and transcription factors such as NF-κB.

#### 4.2.3 Impact on immunotherapy

The gut microbiota appears to shape immunotherapy responsiveness by modulating antigen presentation, effector cell priming, and the balance between immune activation and immune suppression. Commensal bacteria such as *Bifidobacterium* and *Lactobacillus* have been shown to promote DC maturation and antigen-presenting capacity, and CD8+ T cell activation in experimental models. For instance, *Bifidobacterium* supplementation increased intra-tumoral CD8+ T cell infiltration and enhanced the efficacy of immunotherapy in tumor-bearing mice. Furthermore, the gut microbiota may also influence the equilibrium among T cell subsets, including Tregs and T helper 17 (Th17) cells (107–109). Given that Tregs are immunosuppressive cells, and Th17-associated programs may contribute to inflammatory signaling depending on the milieu, microbiota-driven shifts in these populations should be interpreted as immunoregulatory rather than uniformly beneficial or harmful.

The gut microbiota can also influence the TIME through metabolite-dependent and cell-mediated mechanisms, thereby affecting the response to immunotherapies (110–112). Microbiota-derived metabolites, including SCFAs and secondary bile acids, are known to alter cytokine gradients, APC function, activation states of intra-tumoral lymphoid and myeloid populations, and barrier-associated immune homeostasis. SCFAs modulate CD8+ T cell fitness, Treg differentiation, and inflammatory transcriptional programs, whereas secondary bile acids can reshape immune signaling through multiple pathways. In addition, dysbiosis may influence angiogenesis, epithelial stress responses, and tumor-cell metabolism, all of which can secondarily affect immune cell trafficking, retention, and function within the tumor bed (112–114). Accordingly, the current evidence is best interpreted as indicating that the microbiota can condition the immune landscape of colorectal tumors, rather than proving that any single microbial configuration will necessarily enhance immunotherapy response across all clinical settings.

**Figure 5:**
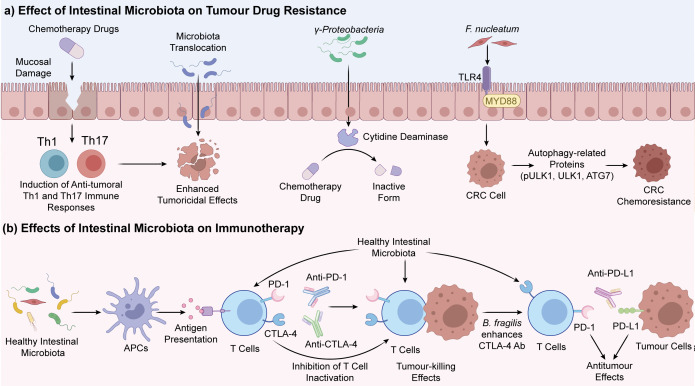
Effects of intestinal microbiota on immunotherapy response. A eubiotic intestinal microenvironment may support APC maturation, antigen presentation, and effector T cell priming, thereby creating a TIME that is more permissive for immune checkpoint blockade therapies **(A)**. In contrast, gut dysbiosis may impair these processes and favor immunosuppressive programs that limit T cell activation. B. fragilis has been shown to enhance the efficacy of CTLA-4 blockade in experimental settings **(B)**.

From a therapeutic perspective, the microbiota-TIME axis is especially relevant to immune checkpoint blockade since checkpoint inhibitors are most effective when the inherent anti-tumor immune response is reinvigorated. A microenvironment enriched in activated DCs, antigen presentation, and cytotoxic CD8+ T cells is generally more compatible with anti-PD-1/PD-L1 or anti-CTLA-4 therapy, whereas accumulation of Tregs, MDSCs, suppressive macrophage states, or poorly functional APCs is more often associated with immune escape and suboptimal benefit. Accordingly, microbiota-directed interventions may influence therapeutic response by altering bacterial abundance, and reshaping DC priming, cytokine tone, lymphocyte trafficking, and the balance between effector and suppressive immune programs. Given the variability in immunotherapy response among individual patients, it will be necessary to incorporate immune cell-specific biomarkers into future microbiota-based therapies. The impact of the gut microbiota on immunotherapy response is depicted in **Figure 5**.

### 4.3 New treatment strategies for CRC based on the gut microbiota

#### 4.3.1 Probiotic intervention

*Bifidobacterium*, *Lactobacillus*, *C. butyricum*, and related strains have been explored as candidate probiotics for reversing CRC-associated dysbiosis. However, the mechanistic basis for these interventions has been primarily elucidated from *in vitro* studies and animal models rather than clinical trials in CRC patients. In experimental settings, *Bifidobacterium* may competitively limit pathogen adhesion and help preserve microecological balance (115–117). In addition, *Lactobacillus* can acidify the intestinal lumen, influence epithelial barrier function, and modulate mucosal immune activity (118–120). Furthermore, *C. butyricum* has been shown to support epithelial integrity and suppress pro-inflammatory signaling by producing high levels of butyrate (121–123). Collectively, these observations provide biological plausibility for probiotic intervention, but should be interpreted as mechanistic and preclinical support rather than direct evidence of established anti-tumor efficacy in clinical CRC care.

In tumor-bearing mice, supplementation with *Bifidobacterium* or *Lactobacillus* has been associated with reduced tumor burden and prolonged survival, indicating that probiotic modulation can influence tumor-related biology under controlled experimental conditions. By contrast, the clinical data on probiotic use for CRC is limited to a few modest cohort studies that have mainly evaluated treatment tolerance, gastrointestinal symptoms, or postoperative recovery, as opposed to oncological endpoints such as objective response, recurrence, or survival (124–126). In addition, the clinical findings are conflicting, which may be attributed to several factors. First, specific strains of *Bifidobacterium*, *Lactobacillus*, and *C. butyricum* may differ in epithelial barrier support, SCFA production, inflammatory signaling, and immune modulation. Second, the dose and formulation of the probiotics determine whether sufficient viable organisms or bioactive products reach the distal intestine and persist long enough to produce measurable biological effects. Third, the timing of the intervention is likely to influence outcomes; for instance, perioperative administration, supplementary use during chemotherapy or radiotherapy, and longer-term supplementation during recovery target distinct biological states and are therefore unlikely to yield identical endpoints. Finally, patients with recent antibiotic exposure, depletion of butyrate-producing commensals, or enrichment of pathobionts may respond differently than those with a relatively preserved microbial ecosystem (144–146). Taken together, the currently available clinical data supports the view that while probiotics are promising adjunctive candidates for supportive care or microbiota modulation, they do not yet justify application as frontline therapy for CRC. Future large-scale, multi-center studies incorporating microbiota stratification and standardized probiotic protocols will be required to determine the clinical utility of probiotic strategies in specific patient subsets.

#### 4.3.2 Prebiotic application

Prebiotics are non-digestible substrates that can be selectively utilized by beneficial intestinal microorganisms. Common prebiotics include fructooligosaccharides, galactooligosaccharides, and inulin. Both experimental and translational studies have shown that these compounds can enrich selected commensal taxa, increase SCFA production, and influence mucosal immune homeostasis (147–149), which in turn may support epithelial health, alter luminal pH, and indirectly restrain expansion of potentially harmful bacteria. In addition, there is evidence that prebiotic supplementation increases the abundance of *Bifidobacterium* and *Lactobacillus*, as well as intestinal immunoglobulin A (IgA) levels (150–152). While these findings indicate biological feasibility, they should not be interpreted as proof that prebiotics independently confer clinically established anti-tumor benefit in CRC patients.

**Figure 6:**
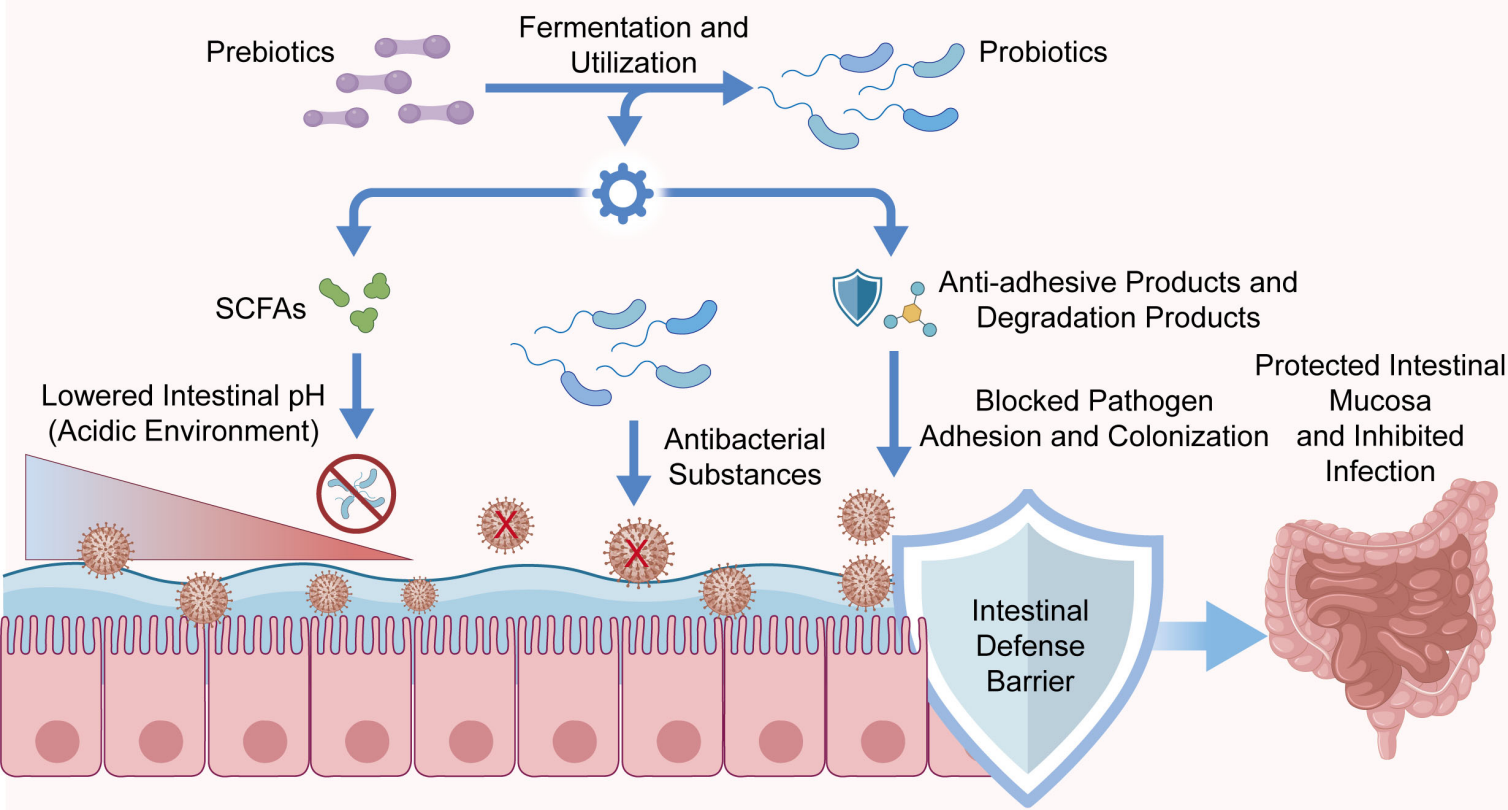
Mechanisms of probiotics and prebiotics against intestinal pathogens. Prebiotics are fermented by probiotics, which produce SCFAs to reduce pH, secrete antibacterial substances, and can also degrade substances to produce anti-adhesion products that prevent pathogens from invading the intestinal mucosa.

The translational role of prebiotics in CRC should be presented with appropriate caution. At present, prebiotics are best regarded as exploratory adjuncts that may help optimize the intestinal environment during chemotherapy, radiotherapy, or immunotherapy, rather than validated efficacy-enhancing interventions. Several studies have reported that prebiotic administration during systemic treatment is associated with less intestinal mucosal injury and lower rates of gastrointestinal adverse events such as diarrhea and nausea (153–155). However, these reports are still limited by small sample size, variable formulations, and the frequent use of intermediate biological or symptom-based endpoints rather than reproducible oncological outcomes. Similarly, although prebiotics may influence intestinal immune function and thereby modulate the response to immunotherapy, direct clinical evidence remains insufficient. Overall, prebiotics warrant further study as microbiota-directed supportive strategies, while definitive conclusions regarding treatment efficacy must await rigorously designed clinical trials (156–158). The mechanisms of probiotics and prebiotics against intestinal pathogens are shown in **Figure 6**.

#### 4.3.3 Fecal microbiota transplantation

**Figure 7:**
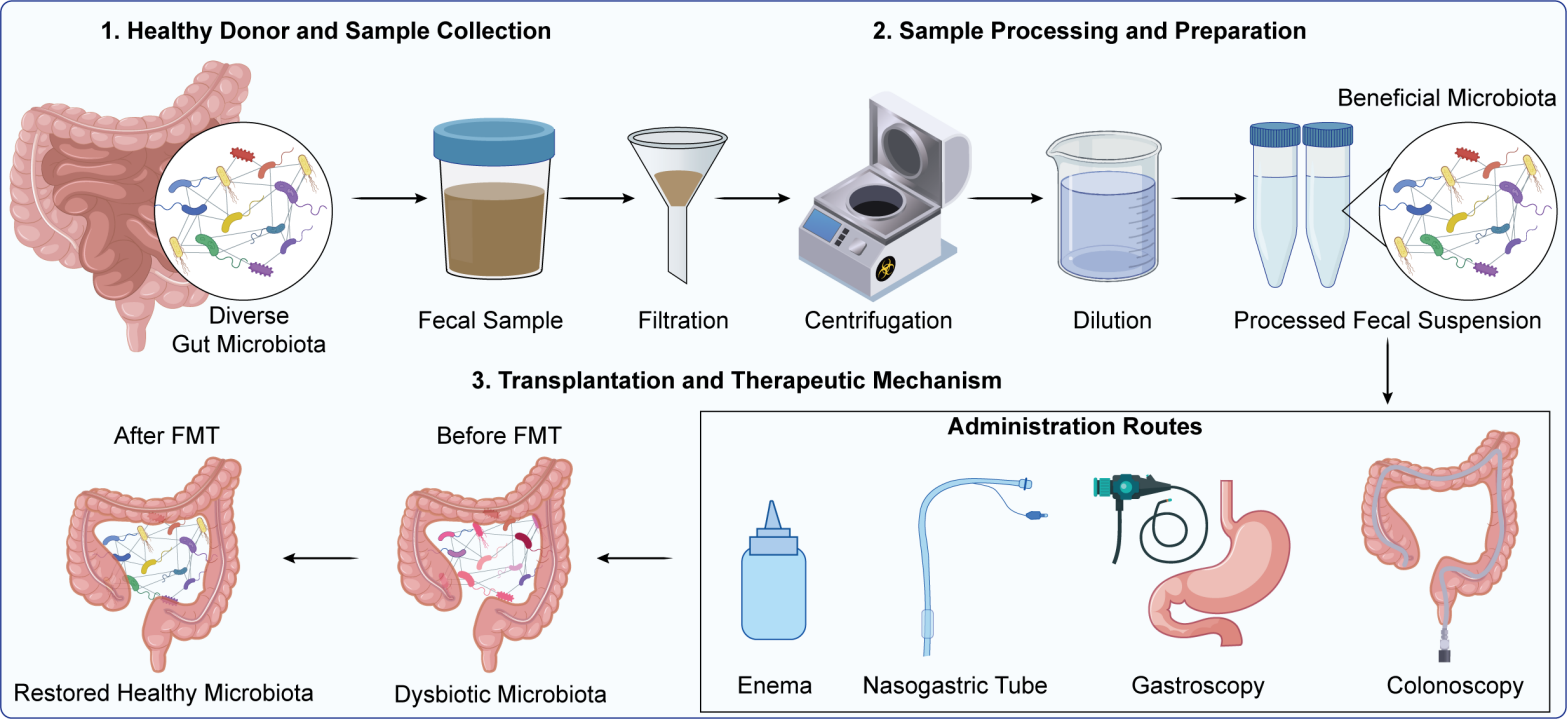
This schematic illustrates the key steps of fecal microbiota transplantation (FMT): stool samples are collected from healthy donors with diverse gut microbiota, processed via filtration, centrifugation, and dilution to prepare a beneficial microbiota suspension, which is then administered to recipients with dysbiotic gut microbiota through routes including enema, nasogastric tube, gastroscopy, or colonoscopy, ultimately restoring a healthy gut microbial profile.

FMT refers to transplanting the fecal microbiota of a healthy donor into a recipient to re-establish the latter’s gut microbiota. Briefly, the feces of a healthy donor is collected, filtered, centrifuged, and diluted to remove impurities and pathogens, and the fecal suspension is transplanted into the recipient’s intestine through colonoscopy (127, 128), gastroscopy, nasogastric tube, or enema (**Figure 7**). The diverse and rich gut microbiota of a healthy donor re-establishes the beneficial bacteria and inhibits the proliferation of harmful bacteria in the patient’s intestine (129–131), thereby restoring the balance and function of the recipient microbiota.

FMT has garnered significant attention for CRC treatment due to its capacity to rapidly remodel the gut microbial ecosystem, and the current evidence suggests that FMT may improve dysbiosis-related intestinal homeostasis and host immune response in selected settings (131–133). Furthermore, proof-of-concept observations from melanoma patients, wherein transfer of fecal microbiota from immunotherapy-responsive donors appeared to restore sensitivity in some previously refractory patients, also support the clinical application of FMT. Nevertheless, these findings cannot be directly generalized to CRC, where tumor biology, treatment context, and host-microbiota interactions may differ substantially. In addition, there are concerns regarding donor selection, stool processing, pathogen transmission, antimicrobial resistance transfer, and recipient safety (134–136). Therefore, given the limited direct evidence of consistent anti-tumor benefit, FMT should currently be considered an investigational microbiota-directed intervention in CRC, which warrants further study under standardized and closely monitored clinical protocols (137–139).

### 4.4 Clinical translation and practical cases of gut microbiota-related research

FMT presents a feasible strategy for reversing CRC-related dysbiosis and potentially modulate host immunity. However, the current evidence from CRC patients is preliminary, and the data are mainly derived from preclinical studies, small exploratory cohorts, or indirect supportive-oncology settings. While proof-of-concept studies in melanoma have suggested that donor FMT may help overcome resistance to immune checkpoint blockade in selected patients, the extent to which these observations can be extrapolated to CRC remains uncertain. In addition, FMT raises important safety concerns, including donor-to-recipient transmission of pathogenic organisms, potential transfer of antimicrobial resistance genes or multidrug-resistant organisms, and unpredictable effects in immunocompromised hosts. To ensure safety and scientific rigor, oncology-related FMT protocols require stringent donor screening, standardized stool processing and storage procedures, longitudinal post-transplant surveillance, and appropriate regulatory oversight. While FMT is promising as an adjuvant microbiota-directed strategy, its clinical value in CRC should be validated in carefully controlled trials before broader application.

#### 4.4.1 Gut microbiota as clinical diagnostic markers for CRC

Given the potential involvement of gut dysbiosis in colorectal carcinogenesis, the key microbial taxa can serve as promising biomarkers for the screening and early diagnosis of CRC. For instance, carcinogenic bacteria such as *F. nucleatum* and pks+ *E. coli* are enriched in the intestinal tissues of CRC patients, whereas probiotics such as *Bifidobacterium* and *Lactobacillus* are relatively depleted. Therefore, the abundance of these characteristic bacteria in the feces or tumor tissues could be useful in differentiating CRC patients from healthy individuals. The intestinal abundance of *F. nucleatum* is related to CRC malignancy and the risk of postoperative recurrence, and its detection sensitivity in feces can reach over 70%. Furthermore, microbiota-derived metabolites such as SCFAs and secondary bile acids are also promising diagnostic indicators of colorectal carcinogenesis. A decrease in butyric acid levels is associated with intestinal mucosal barrier damage, while an increase in pro-inflammatory bile acids such as deoxycholic acid indicates a deterioration of the tumor microenvironment. Therefore, a multi-dimensional diagnostic model can be constructed by integrating microbiota metabolomics with conventional biomarkers to improve the detection rate of early-stage CRC. The direct comparison of microbiota-based biomarkers with established CRC screening tools is summarized in **Table 1**. Although microbiota panels are stool-based and minimally invasive, their specificity, routine implementation, and cost structure remain less standardized compared with fecal immunochemical testing, multitarget stool DNA, and colonoscopy. Accordingly, microbiota-derived markers are currently interpreted as complementary tools for early risk stratification, biological enrichment, or combined-model development, rather than as stand-alone replacements for established CRC screening protocols.

**Table 1:** Direct comparison of microbiota-based markers and conventional CRC screening tools.

Modality	Sample/procedure	Representative specificity	Relative cost	Invasiveness	Current role/key limitation
Fecal microbiota markers (single taxa or multi-taxon panels)	Stool; qPCR, 16S rRNA, or metagenomic analysis	Variable and cohort-dependent; promising in discovery/validation studies, but no universally standardized specificity threshold for routine screening	Moderate to high	Low/noninvasive	Promising adjunct for risk stratification or triage; limited by inter-cohort reproducibility, pre-analytical variation, bioinformatics standardization, and assay harmonization
FIT	Stool hemoglobin immunoassay	Approximately 93-94%	Low	Low/noninvasive	Established first-line noninvasive screening tool; requires repeated testing and has lower sensitivity for some advanced precursor lesions
mt-sDNA/sDNA-FIT	Stool DNA plus hemoglobin-based assay	Approximately 85-89%	Moderate to high	Low/noninvasive	Higher sensitivity for CRC than FIT in many studies, but more false positives and higher cost
Colonoscopy	Endoscopic visualization with bowel preparation	Approximately 86-89% in comparative screening analyses	High	High/invasive	Reference-standard examination with biopsy and polypectomy capability, but limited by bowel preparation, sedation, complications, and lower acceptability in some populations

#### 4.4.2 Clinical cases of gut microbiota-targeted therapy

Early reports suggest that FMT may improve microbial composition and support intestinal recovery or treatment responsiveness in CRC patients in selected settings. However, the currently available studies are based on small and heterogeneous cohorts, and are often exploratory in design. There is evidence of occasional tumor shrinkage post-FMT in melanoma patients resistant to immune checkpoint inhibitors, but these observations are derived mainly from proof-of-concept experience and cannot yet be translated into a reliable remission increment for CRC. Likewise, reports of shorter postoperative recovery time or fewer infectious complications after FMT should be interpreted cautiously unless sample size, comparator structure, and statistical significance are explicitly reported and reproduced in adequately powered trials.

Clinical studies evaluating the combination of probiotics (*Bifidobacterium*, *C. butyricum*) and chemotherapy suggest that microbiota modulation may influence the intestinal immune milieu and treatment tolerance. Supplementation with *C. butyricum* has been associated with enhanced 5-FU-induced apoptosis and lower expression of drug-resistance pathways in preclinical models, but clinical confirmation remains limited. Furthermore, probiotic intervention has been shown to alleviate gastrointestinal adverse effects such as nausea and vomiting, and improve patient-reported quality of life in small clinical trials, although the magnitude of benefit varies depending on the formulation and study design. Prebiotics (such as fructooligosaccharides) may also indirectly restrain tumor-promoting conditions by enriching beneficial bacteria, lowering luminal pH, and suppressing harmful colonization. Some studies have reported increased gut microbiota diversity and lower levels of IL-6 and other inflammatory markers after prebiotic supplementation in postoperative or adjuvant settings; however, these findings should be interpreted in light of sample size, randomization status, endpoint definition, and whether inter-group differences reached statistical significance.

Clinically, personalized microbiota-directed CRC treatment should ideally be approached in a stratified rather than “one-size-fits-all” manner. Patients with distinct microbial features, host immune states, and treatment contexts are unlikely to benefit similarly from the same intervention. For example, individuals with *F. nucleatum* enrichment and ongoing chemotherapy may represent a subgroup in which targeted antimicrobial strategies or microbiota-modulating combinations could be explored, whereas metabolite-restoring probiotic or prebiotic approaches aimed at supporting mucosal homeostasis and treatment tolerance may be more suitable for patients with depletion of butyrate-producing commensals, treatment-related mucosal injury, or marked barrier dysfunction. Similarly, microbiota-based interventions aimed at enhancing antigen presentation, DC priming, or effector cell function in patients undergoing immunotherapy are unlikely to be equally relevant across immune-inflamed, immune-excluded, and strongly suppressive tumor milieus. It is possible to classify host immune phenotypes by integrating microbial composition, functional pathways, metabolite profiles, host transcriptomic features, immune phenotypes, and clinical variables using machine-learning algorithms such as Random Forest and XGBoost. These models may help stratify patients based on recurrence risk, predict therapeutic response, and identify patients more likely to benefit from specific microbiota-directed interventions. For clinical interpretability, future studies should report standardized performance metrics, including the area under the receiver operating characteristic curve (AUC) for classification tasks and the concordance index (C-index) for survival or recurrence-related endpoints.

#### 4.4.3 Challenges and countermeasures in clinical applications

Although FMT may restore the gut microbiota in selected CRC patients with profound dysbiosis, and alleviate treatment-related intestinal toxicity, its clinical translation still requires prospective validation under carefully monitored clinical protocols. At present, FMT is better viewed as an exploratory or investigational intervention in rather than a standard microbiota-targeted therapy. The long-term effects of probiotics or FMT are also not clear, and potential risks such as microbiota imbalance or the transfer of drug resistance genes need to be monitored. Large-scale clinical studies with long-term follow-up are required to establish an early warning mechanism for adverse reactions. Gut microbiota-related treatments involve multiple fields such as gastroenterology, oncology, and microbiology. Therefore, it is necessary to strengthen interdisciplinary cooperation and develop standardized diagnosis and treatment guidelines.

#### 4.4.4 Frontiers and prospects of clinical research

Currently, multiple clinical studies are exploring FMT and other gut microbiota-targeted strategies in CRC, and the combination of these approaches with immunotherapy in selected populations, such as patients with high microsatellite instability. While these studies are a step to clinical translation, they should still be interpreted primarily as feasibility and signal-generating efforts until efficacy estimates are supported by clearly reported patient numbers, comparator structures, and formal statistical analysis. Imminent advances in single-cell sequencing, gene editing, and multi-omics integration may help refine microbiota-informed precision medicine for CRC. Furthermore, Random Forest, XGBoost, or related ensemble-learning approaches may facilitate the integration of metagenomic, metabolomic, transcriptomic, and immune cell-resolved data into clinically actionable models. Beyond reporting response rates alone, these precision-medicine studies should ideally be evaluated using discrimination and calibration measures such as AUC, C-index, calibration plots, and external-validation performance to improve their translational relevance.

The heterogeneity of the gut microbiota across individuals leads to uneven treatment effects. For FMT in particular, donor screening criteria, manufacturing consistency, route of administration, and recipient selection have not yet been fully standardized. In future, it will be necessary to establish an individualized assessment system based on host genes, diet, and microbiota characteristics, and to optimize treatment plans by combining machine learning with standardized safety frameworks. Furthermore, the long-term effects of probiotics or FMT are not yet clear, and potential risks such as microbiota imbalance, pathogen transmission, and the transfer of drug-resistance determinants require continued monitoring. In addition, because oncology-related FMT remains largely investigational outside a few narrowly defined indications, its clinical application should proceed under appropriate ethical and regulatory oversight, with rigorous informed consent, traceability, and quality-control procedures. Large-scale, long-term follow-up studies are required to establish an adverse-reaction early-warning mechanism, and to define the regulatory conditions necessary for broader translation. Despite these challenges, the gut microbiota has emerged as a valuable entity in the diagnosis, treatment, and prognosis of CRC. In the future, high-quality clinical research and standardized practices are needed to accelerate the promotion of microbiota-targeted therapies as an important part of the comprehensive treatment of CRC.

## 5 Research status and challenges

### 5.1 Summary of the main research achievements

The role of the gut microbiota in the occurrence and treatment of CRC has been elucidated in greater detail in recent years, with many studies demonstrating an association between gut dysbiosis and CRC (140–142). Furthermore, the composition of the gut microbiota of CRC patients and healthy people shows significant differences. For example, pathogenic bacteria such as pks+ *F. nucleatum* and *E. coli* are enriched, while beneficial bacteria such as *Bifidobacterium* and *Lactobacillus* are reduced in CRC patients. The gut microbiota may influence colorectal tumorigenesis through genotoxic effects, inflammation-mediated mechanisms, harmful metabolites, and immune dysregulation (107, 108). However, the current evidence largely supports association and biological plausibility rather than definitive causation in humans. Colibactin-producing pks+ *E. coli* and enterotoxigenic *B. fragilis* may directly damage the DNA of intestinal epithelial cells or remodel the inflammatory microenvironment in experimental settings. Furthermore, harmful metabolites produced by the gut microbiota, such as hydrogen sulfide and secondary bile acids, have cytotoxic and pro-inflammatory effects (109–111), while the reduction in beneficial metabolites like SCFAs may weaken epithelial protection and tumor-suppressive immune regulation. Gut dysbiosis can also be accompanied by abnormal activation and differentiation of immune cells, potentially affecting immune surveillance and facilitating tumor progression.

In addition, the gut microbiota may influence treatment response in CRC, although the evidentiary strength differs across experimental systems and clinical settings. Microbiota-dependent effects on drug metabolism, mucosal injury, immune tone, and treatment tolerance are supported by a substantial body of mechanistic studies. For example, *F. nucleatum* has been linked to oxaliplatin and 5-FU resistance in experimental studies [180,181], and *C. butyricum* has been reported to enhance the activity of 5-FU in preclinical models. Likewise, probiotics such as *Bifidobacterium* and *Lactobacillus* may promote mucosal repair and modulate inflammatory responses after radiotherapy [182–184], while microbiota-derived metabolites may influence the TIME during immunotherapy. However, these findings do not conclusively prove that microbiota-targeted interventions can improve major oncologic endpoints in patients. At present, the most definitive clinical implications relate to treatment tolerance, microbiota remodeling, and patient stratification, whereas durable efficacy benefits still require confirmation through standardized prospective trials.

### 5.2 Problems and challenges in research

Despite the significant body of research on the gut microbiota and CRC, there are considerable limitations and challenges. Although some CRC-specific microorganisms such as *F. nucleatum* and pks+ *E. coli* have been discovered (112–114), it is still impossible to determine a single carcinogenic microorganism. The occurrence of CRC may be the result of the combined action of multiple microorganisms and their metabolites. In addition, the interaction and synergy between these microorganisms, and their carcinogenic mechanisms need further study. Furthermore, most studies on the gut microbiota and CRC are cross-sectional or case-controlled analyses, which makes it difficult to determine whether gut microbiota dysbiosis is a cause, a consequence, or simply a coexisting feature of CRC. In fact, the gut microbiota can be affected by colorectal carcinogenesis, dietary patterns, antibiotic exposure, inflammation, and treatment, which increases the risk of causal misinterpretation (115, 116). To address this limitation, future studies should integrate longitudinal cohort designs with serial biospecimen collection before adenoma or CRC onset, thereby enabling temporal assessment of microbial shifts. In addition, gnotobiotic models colonized with defined strains or synthetic microbial communities can be used to directly test causality and mechanism under controlled conditions. Combining these approaches with carefully controlled interventional studies will be critical for strengthening causal inference.

### 5.3 Prospects for future research directions

There are several research directions that need attention for extending the current information on the relationship between the gut microbiota and CRC into clinical applications. First, causal inference should be strengthened through well-designed longitudinal cohort studies with repeated sampling across the adenoma-carcinoma sequence, along with rigorously controlled animal experiments, gnotobiotic models, and mechanistic *in vitro* tests, so as to clarify whether gut microbiota dysbiosis precedes, accompanies, or follows colorectal carcinogenesis (117, 118). In particular, gnotobiotic systems colonized with defined strains or synthetic communities can help determine whether candidate microorganisms are sufficient to modulate tumor initiation or progression, and can provide a more direct connection between association and mechanism. Second, gene editing and single-cell sequencing technologies can be applied to explore the interaction mechanisms between the gut microbiota and host cells, and identify the key signaling pathways and molecular targets by which the gut microbiota affects CRC occurrence (119–121) and development. In this context, single-cell sequencing, spatial transcriptomics, and multiplex immune profiling should be systematically incorporated into future studies to define how microbial dysbiosis relates to specific TIME phenotypes. These strategies may help identify microbiota-associated immune programs, including exhausted CD8+ T cells, Treg/MDSC enrichment, and myeloid-inflammatory niches, thereby improving mechanistic interpretation and facilitating patient stratification for microbiota-modulating or immunotherapy-based interventions.

Personalized gut microbiota intervention is also a promising endeavor [195–197], one that will require patient stratification based on baseline dysbiosis pattern, treatment setting, host immune phenotype, and safety-related context as opposed to generic microbiota supplementation. Patients with treatment-related mucosal injury or antibiotic-exposed dysbiosis during chemotherapy or radiotherapy may be better suited to supportive microbiota-restoring strategies, whereas patients being considered for immunotherapy may need to be screened according to microbial features linked to antigen presentation, DC priming, and T cell function. Likewise, FMT may be more appropriate for carefully selected patients with profound dysbiosis under tightly regulated protocols, while targeted probiotic or prebiotic interventions may be preferable when the therapeutic goal is restoration of butyrate production, mucosal repair, or restraint of tumor-promoting inflammation. Due to the significant impact of individual differences on the gut microbiota, it is necessary to implement personalized intervention strategies based on the genetic backgrounds, dietary structures, lifestyles, and the composition and functional characteristics of the gut microbiota [198–200]. Multi-omics methods such as metagenomics, metabolomics, and transcriptomics will be helpful in evaluating the characteristics and functions of the individual gut microbiota, which in turn will allow screening of the best probiotics, prebiotics, and FMT donors to achieve optimal outcomes. It is also worthwhile to explore the efficacy of combining FMT and immunotherapy [201–203], and different probiotics and prebiotics, to improve the efficacy of gut microbiota intervention.

One of the future research directions is the exploration of gut microbiota-related biomarkers associated with the occurrence, development, and treatment response of CRC, which can be harnessed for the early diagnosis, prognosis, and treatment of CRC. Artificial intelligence, big data technology, and multi-omics data can be integrated to establish a prediction model for the relationship between the gut microbiota and CRC (122–124), so as to guide clinical decision-making. It is also imperative to establish unified research methods, quality control standards, and clinical application guidelines to accelerate the transformation of research results related to the gut microbiota (125, 126, 143). For example, Random Forest and XGBoost provide representative model architectures that can capture non-linear interactions among microbial, metabolic, host, and immune features, making them attractive for patient stratification and therapeutic decision support. In future personalized-therapy studies, model performance should be reported in a standardized manner, with AUC used for diagnostic or treatment-response prediction tasks and C-index applied to time-to-event outcomes such as recurrence-free or overall survival; calibration assessment and external validation will also be essential to reduce overfitting and improve generalizability.

## 6 Conclusion

In summary, four evidence-based conclusions emerge from the current literature. First, gut dysbiosis is consistently associated with CRC across experimental and clinical settings. However, the clinical data supports association, temporal linkage, and biological plausibility rather than definitive causation. Second, specific microorganisms and their products may participate in colorectal tumorigenesis through genotoxic stress, chronic inflammation, metabolic remodeling, and immune dysregulation within the tumor microenvironment, although the relative contribution of each pathway is likely context dependent. The aim of this review was not merely to catalogue individual microorganisms or pathways, but to explore the impact of the gut microbiota on immune and metabolic programs relevant to colorectal tumorigenesis and therapeutic responsiveness. Third, the gut microbiota influences not only tumor initiation but also treatment response by regulating the metabolism of chemotherapeutic drugs, radiation-associated mucosal injury, and the tumor immune landscape. Fourth, microbiota-directed strategies, including probiotics, prebiotics, and carefully regulated FMT, remain promising but should still be regarded as investigational approaches until they are supported by standardized, adequately powered, and clinical endpoint-driven studies. Taken together, the available data are most usefully synthesized through a microbiota-immune-metabolism-therapy axis, in which ecological disruption, immune contexture, metabolic signaling, and treatment susceptibility are viewed as interdependent but not yet fully disentangled components of CRC biology.

Several testable hypotheses should guide future work. One hypothesis is that defined microbial consortia enriched in *F. nucleatum*, pks+ *E. coli*, or enterotoxigenic *B. fragilis* is sufficient to accelerate adenoma-to-carcinoma progression in gnotobiotic models, whereas restoration of butyrate-producing communities may attenuate this trajectory. A second hypothesis is that microbiota configurations associated with CD8+ T cell exclusion and Treg/MDSC enrichment correlate with, and may even predict, inferior response to immune checkpoint blockade, thereby guiding patient stratification. A third hypothesis is that multi-omics models integrating metagenomic, metabolomic, transcriptomic, and immune cell-resolved features may outperform single-modality biomarkers for recurrence prediction and treatment selection when assessed by discrimination, calibration, and external validation. Addressing these hypotheses through longitudinal cohorts with serial biospecimen collection, mechanistic intervention studies, and harmonized analytical pipelines will be essential for moving microbiota-based precision oncology from conceptual promise toward clinically actionable practice in CRC.
